# Association between Smoking and Sugar-Sweetened Beverage Consumption, Tooth Brushing among Adolescents in China

**DOI:** 10.3390/children9071008

**Published:** 2022-07-06

**Authors:** Haihua Zhu, Huan Zhou, Qin Qin, Weifang Zhang

**Affiliations:** Stomatology Hospital, School of Stomatology, Zhejiang University School of Medicine, Zhejiang Provincial Clinical Research Center for Oral Diseases, Key Laboratory of Oral Biomedical Research of Zhejiang Province, Cancer Center of Zhejiang University, Hangzhou 310006, China; zhuhh403@zju.edu.cn (H.Z.); huanzhou87@zju.edu.cn (H.Z.); qinqin@zjkq.com.cn (Q.Q.)

**Keywords:** adolescent smoking, sugar-sweetened beverage, tooth brushing, oral health-related quality of life

## Abstract

The study aimed to investigate the relationship between smoking, Sugar-Sweetened Beverage (SSB) consumption and tooth brushing among adolescents in China. A valid sample of 6084 middle school students from the Zhejiang province was included. Participants were questioned about smoking status, SSB consumption, tooth brushing, and oral health-related quality of life (OHRQoL). Among the participants, smoking prevalence was 1.9% and nearly half of the students consumed SSBs. The demographic factors associated with smoking were gender, place of residence, and parental level of education. There are co-variations between smoking status, SSB consumption, and tooth brushing. Logistic regression showed that smoking adolescents were more likely to brush their teeth less than once per day (OR = 1.74, *p* < 0.05), consume soft drinks once or more per day (OR = 2.18, *p* < 0.01) and have a higher score on the Child Oral Health Impact Profile (OR = 1.05, *p* < 0.05) after adjusting for demographic factors. The findings provide compelling evidence for governments and related stakeholders to intervene in the lifestyle of adolescents. Future studies are needed to understand the interaction effects of such behaviors, and should help to inform appropriate interventions.

## 1. Introduction

Adolescence, as a critical period in life, has significant biological and psycho-social characteristics which may compromise or affect health in the long run [1]. It is also a key time to avoid related risk factors which may foster adverse health outcomes. Most studies about adolescents focus on behaviors such as tobacco use, excessive alcohol intake, and sexual and injury-related behaviors [2]. Among these behaviors, tobacco has caused a significant threat to global public health. More than 8 million people die of tobacco-related diseases each year around the world [3]. These people typically start smoking as teenagers and become more dependent on nicotine in adulthood [4]. Additionally, peer influence, such as sharing cigarettes among adolescents, might increase the possibility of smoking initiation or relapse [5]. Nicotine addiction affects cognition and adolescents’ brains are more sensitive to nicotine. Therefore, smoking during adolescence is more likely to cause cognitive impairment in adulthood [6]. Moreover, smoking at an earlier age could increase the risk of developing illness and early death, particularly cancer, cardiovascular and pulmonary diseases [7]. Sugar-sweetened beverage (SSB) consumption among children and adolescents presents a global healthcare problem. SSBs are the main sources of free sugar intake, or sugars added to food and drinks, including milk, tea, and coffee. SSBs account for 69% of added sugars in Mexico [8]. A high level of free sugar intake is a primary pathogenic factor of systemic diseases such as obesity [9,10], cardiovascular disease (CVD) [11], metabolic syndrome, and so forth. Oral diseases are among the most prevalent non-communicable diseases (NCDs) globally [12]. Tobacco use could trigger the development of oral cancer [13]. Sugar in food and drinks could increase the morbidity rate of caries or periodontal disease in adolescents [14,15]. Thus, they are modifiable risk factors for oral diseases. Considering the WHO’s definition of health as a state of complete physical, mental, and social well-being [16], oral health is a state which enables individuals to eat, communicate, and fully participate in their chosen social roles. Locker et al. introduced the concept of oral health-related quality of life (OHRQoL) to measure oral health status. Defined by individual assessment of several oral health dimensions that affect overall well being [17], OHRQoL was widely used over the past two decades [18]. Reported information quantifying the effects of smoking on OHRQoL is inadequate [19], especially among adolescents. This study also measured OHRQoL to reveal smoking’s direct impact on oral health and prove that smoking is an oral health-related behavior.

Poor oral health-related behaviors are usually indicative of tobacco use, a high level of sugar intake, and infrequent tooth brushing. All of which can harm adolescents’ physical and psychological health and could be seen as adolescent health risk behaviors or problem behavior syndrome. The extant research from the perspective of problem behaviors/health risk behaviors of adolescents often focuses on precocious sexual intercourse, problem drinking, and violence. Few researchers have investigated the relations between more than two behaviors [20,21]. Various problem behaviors were interrelated, found to have similar determinants, and fulfill similar functions [22]. This study provides a new perspective on oral health-related behaviors.

Previous studies on tobacco use, high sugar intake, and tooth brushing considered these behaviors as factors related to the oral clinical outcome; however, none of such studies explored the relationship of the three behaviors from the perspective of problem behavior theory. With this background, the present study was designed to find the association between smoking, SSBs, and tooth brushing, and to explore whether smoking adolescents have a lower level of oral health-related quality of life by controlling for related factors.

## 2. Materials and Methods

### 2.1. Participants and Procedures

We used a cross-sectional multistage systematic to select the representative study sample. Located in southeast China, with a population of 60.7 million, Zhejiang Province has 11 cities and 90 districts or counties. In this study, we randomly selected three residential districts and three counties. Three of the residential middle schools were selected in the main urban area of each district and county using the Probability Proportional to the Sampling size (PPS) method. Finally, adolescents aged 12–15 years old attending these middle schools were recruited using a quota sampling method. The following formula was used to calculate the required sample size:

*n* = *deff (*µ^2^*p*(1 − *p*)/ε^2^), (1)

In the formula, *n* is the sample size, *deff* is the design effect, set as 4.5, *p* is the dental caries prevalence (28.9%), μ is the level of confidence, and ε is the margin of error. The non-response rate was 5%. Based on this estimation, the final sample size was 4485 [23].

A written questionnaire that included social demographic characteristics was scheduled. After being told the survey’s purpose, participants completed the questionnaire within approximately 20 min in a private and quiet environment. The research group promised all participants that their school teachers, schoolmates, parents, and other family members would never know their answers. The study protocol was approved by the Stomatological Ethics Committee of the Chinese Stomatological Association (No.2014-003). Written informed consent was obtained and all participants were given permission by their guardians.

### 2.2. Measures

For social demographic characteristics, gender, age, ethnicity (There are 56 ethnic groups, each ethnic group is coded), only child status, and parental education level were included. Regions were categorized into rural/urban areas according to the administrative area code.

Participants were asked whether they were current smokers and how often they smoked. Daily smokers were those who smoked every day; weekly smokers were those who smoked several times a week; occasional/former smokers were those who smoked occasionally or had smoked before [24]. SSB consumption was valued by the frequency of soft drink consumption and the intake of sugary milk, tea, and coffee. Responses ranged from ‘seldom/never’, ‘1–3 times per month’ to ‘twice or more than twice per day’, and were coded as 1, 2 through 6, respectively. Oral hygiene behavior, valued by tooth brushing, asked if respondents brushed teeth and how frequently.

For the oral health-related quality of life (OHRQoL), a simplified Chinese version of the child oral health impact profile (COHIP) was used. The COHIP was developed to assess children’s oral well-being withing a wide age range (8–15 years) and across ethnicities, health systems, and various conditions, and was translated with a Chronbach’s alpha of 0.81 [25,26,27]. The simplified Chinese version of COHIP was widely used in the Fourth National Oral Health Survey of China. It includes nine items to capture four dimensions: ‘Functional well-being’ (three items including trouble pronouncing words, discomfort when eating and brushing teeth); ‘Psychological well-being’ (two items including easily being worried and unsatisfactory sleep); ‘School/Environment’ (two items including avoiding going to school and doing housework); ‘Self-image and Social well-being’ (two items including avoiding smiling and social communication) [28]. Each item is assessed using the same question ‘how greatly has it affected you during the past six months because of your oral problems’ answered on an ordinal scale from 4 to 1 (‘severe’, ‘median’, ‘minor’, ‘none’) (Table 1). A total score is obtained by adding up the points for the individual questions.

### 2.3. Statistical Analysis

Data were entered into a database using Microsoft Excel and then imported into SPSS (V.25.0) and Jamovi 1.8.1 for statistical analyses. Frequencies were calculated to summarize the distributions of the categorical variables. We performed Chi-square testsand Fisher’s chi-squared tests to examine the distributions of the categorical behavioral variables (smoking, SSB consumption, oral health behavior) with demographic factors. The associations between smoking and OHRQoL were tested by Mann–Whitney U tests. Binomial logistic regression models were used to calculate odds ratios (ORs) and 95% confidence intervals (CIs) with smoking status as a dependent variable for estimating associations between smoking, SSB consumption, and oral health behaviors (model 1–4). Binomiallogistic regression models were used to calculate ORs and 95% CIs with smoking status as a dependent variable. All models above were controlled for demographic factors. Statistical tests were two-sided, and statistical significance was set at *p* < 0.05.

## 3. Results

Out of the total sample (6480 individuals), 6307 (97.3%) were successfully contacted and agreed to participate in the study. Among the total sample, 49.8% were male. The majority were of the Han nationality. 44.8% of the participants were the only child in their family, 60.8% of their fathers, and 65.1% of their mothers did not receive a high school education (Table 2).

### 3.1. Factors Associated with Smoking

The daily smoking prevalence was 0.1%, weekly smoking prevalence was 0.2%, and 1.6% were occasional or former smokers. More boys (2.6%) than girls (1.1%) reported smoking. Smoking prevalence was associated with gender, place of residence, parents’ highest education level, frequency of tooth brushing, and SSB consumption (Table 2). 2.6% of boys and 1.1% of girls reported smoking. Among the smokers, more of them were from urban cities (2.2%). Additionally, 2.9% of adolescents whose fathers had only elementary school degrees smoke much higher than other groups. The smoking prevalence of participants with different tooth-brushing frequencies was 3.0% (less than once per day), 1.8% (once per day), and 1.4% (twice or more per day). The smoking prevalence of participants increased as the frequency decreased.

### 3.2. Social Demographic Factors Associated with SSB Consumption and Oral Health Behavior

A total of 2827 (45%) reported soft drink consumption twice or more per week. The frequency of soft drink consumption was associated with age, gender, nationality, only child status, and father’s highest education level. Students who were older than 13 years old consumed more soft drinks. 53% of boys and 38% of girls reported soft drinks consumed more than once per week. Minorities preferred such drinks more than did those of the Han nationality. Students with status as an only child seemed to consume fewer soft drinks. The higher the father’s education, the less frequently the children drank soft drinks.

Participants drinking sugary milk, tea, or coffee ‘more than once per day’ and ‘2–6 times per week’ comprised 34.9% and 26.6%, respectively. There was no difference between social demographic factors and the frequency of sugary drink consumption (Table 3). After a simple field investigation, we found that sugary milk tea, tea drinks, and sweetened milk beverages were the most common drinks among middle school students, especially bubble tea.

More than half of the participants brushed their teeth less than twice per day, and 20.3% did not brush their teeth every day (Table 2). The frequency of tooth brushing was associated with all social demographic factors (Table 3). Adolescents 14 years old, male, national minority, non-only-child, and with less-educated parents were more likely to brush their teeth less than twice per day or consume more SSBs than others.

### 3.3. Smoking and SSB Consumption & Oral Health Behavior

We adjusted demographic characteristics viewed separately in the applications of models 1–3. The full logistic regression model for the smoking situation found that frequency of tooth brushing and soft drink consumption were prominent predictors of tobacco use. Smoking adolescents were more likely to brush their teeth less than once per day (OR = 1.74, 95%-CI: 1.03, 2.93, *p* < 0.05) and consume soft drinks once or more per day (OR = 2.18, 95%-CI: 1.32, 3.60, *p* < 0.05) (Table 4).

### 3.4. Smoking and OHRQoL

The median score of COHIP was 5 (3, 6) (Table 2). The smokers reported significantly lower scores (*p* < 0.01) (Table 2), with the different frequency distribution of responses in pronouncing words (*p* < 0.01), easily being worried (*p* < 0.01), sleeping (*p* < 0.05), smiling (*p* < 0.01), social communication (*p* < 0.01), going to school (*p* < 0.01) compared to nonsmokers. There was no difference in eating, doing housework, and brushing teeth (Table 5). Multiple logistic regression shows that smoking adolescents were more likely to have a higher score of COHIP (OR = 1.05, 95% CI: 1.00, 1.09, *p* < 0.05) by adjusting demographic factors, frequency of tooth brushing and SSB consumption.

## 4. Discussion

According to the WHO report, around 6% of adolescents aged 13–15 years old report smoking cigarettes (8% of boys and 3% of girls) worldwide [3]. In Hongkong, in 2017, the smoking prevalence within 30-days of adolescents (school students) was 2.5% [29]. In our study, however, about 1.9% of participants had used cigarettes, a number much lower than those results. The characteristics of middle school students might be the reason. China remains the largest tobacco producer in the world, and has more smokers than any other country [30]. One in eleven adolescents has smoked tobacco in China [31]. Given that our participants were 12–15 years old, results showed that most Chinese smokers started smoking above the age of 15 years old [32], much older than those from other countries. In addition, the anti-smoking regulations and the traditional view that smoking is harmful to health in Zhejiang province could also be major influencing factors. The local government has set up other anti-tobacco measures, including the placement of anti-smoking advertisements, restricting minors from buying cigarettes, setting up smoke-free regulations, promoting smoke-free households/offices/campuses, and carrying out tobacco control courses since 2010 [33]. In 2021, the government implemented the Notice on Further Strengthening the Construction of Smoke-free Schools formulated by the National Health Commission of the People’s Republic of China which prohibits smoking in all schools, including primary school, middle school, and colleges [34]. According to the China Report on the Health Hazards of Smoking 2020, the Zhejiang province maintains a low smoking prevalence among adults compared to other provinces [35].

The US Nutrition Examination Survey showed that 64% of youth aged 2–19 years consume SSB daily [36]. Nearly half of the youth aged 2–18 years old drink an average of 217 mL of SSBs per day in Australia [10]. In our study, nearly half of the students consumed SSBs, while the frequency of soft drink consumption was higher than that of sugary milk, tea, and coffee (45% and 62%). Another study in China also showed that 66.6% of participants aged 6–17 years consumed SSBs [37]. High frequency of free sugar intake could be seen as an eating disorder, which leads to numerous anomalies in the oral cavity and affects both the teeth and soft tissues, and finally causes oral mucosa injuries, diseases of the periodontium, and dental decay [38]. Soft drink consumption was associated with some social demographic factors, but sugary milk, tea, and coffee consumption were not. This kind of association caused much concern about SSBs, including bubble tea, herbal tea, and tea/milk beverage. They all had free sugar. For example, the sugar contained in one cup of 16-ounce boba drink (a common type of bubble tea) exceeds the upper limit of added sugar intake recommended by the 2015 US Dietary Guidelines Advisory Committee [39]. With an affordable price, sweet flavors, and multiple choices, bubble tea has become highly attractive to teenagers willing to try new things. Although bubble tea contains tea and milk, its sugar content is much greater than that of real tea or milk. Moreover, in addition to sweet bubble tea liquid, the ingredients may also have a great deal of sugar, a fact ignored by many teenagers and their parents. Some merchants claim that their bubble tea is “nutritious” or “healthy” in order to expand their customer base.This attractive advertisement cheats parents and even educators: some middle schools provide sweetened milk beverages in student lunches instead of fresh milk or soybean milk. Other countries also have the same issue [40,41], but China’s attention to this situation is far less than that of other countries.

Brushing teeth twice daily with the use of fluoride toothpaste and a concentration of 1000 ppm or above is recommended for adolescents [42]. In our study, only 41% of participants brush their teeth twice a day. Male, minority, children with siblings, and those with less-educated parents were more likely to have irregular oral hygiene habits. Our result is similar to previous studies [43]. Poor oral hygiene status significantly increasesthe likelihood of gingival bleedingand dental caries among children [44,45].

This study explored the relations between smoking, SSB consumption, and tooth brushing in China. After adjusting for demographic factors, the relevance still exists. This relevance illustrates deeper connections between smoking, SSB consumption, and oral health behaviors. Given the study’s cross-sectional design, causation cannot be inferred from the associations identified. While corresponding factors such as mental stress, adolescent smoking, and depression have also been explored [46], adolescents with attention-deficit/hyperactivity disorder (ADHD) are at increased risk of initiating tobacco use at an earlier age [47]. The association between SSB consumption, addiction, and depression is also widely reported [48]. Multiple logistic regressions also show that smokers had poorer OHRQoL compared to non smokers, which is similar to the findings of former studies [49,50]. The interaction effects of these behaviors and the observed associations on whether negative feelings work as a third variable still require further research and investigation.

Health-related quality of life was defined as ‘the functional effects of an illness and its consequent therapy upon a patient, as perceived by the patient’ by Schipper [51,52], and evidence shows that both passive and active smoking are associated with chronic rhinitis in children [53], and impact the children’s health-related quality of life [54]. The results of our study showed that smoking mainly has negative effects on adolescents’ psychological and social health, while there was no difference in functional well-being such as eating or brushing teeth. Thus, we should pay attention to smoking adolescents’ mental health and social function. Interdisciplinary studies with mature theory should be conducted for special populations. These theories could broaden the research horizon and deepen the understanding and knowledge of oral health. Our study used Problem Behavior and the results suggest its application was appropriate. Smoking, toothbrushing, and SSB consumption have intra-individual linkages and co-occur within the same adolescent. Thus these behaviors might be an organized constellation of behavior rather than being a collection of independent, discrete activities. They could be seen as adolescent health risk behaviors or problem behavior syndrome. Understanding their origin, nature, and impacts on health across the entire life span is important. Health risk in adolescence can refer to a risk that is either immediately consequential within adolescence or has consequences for the post-adolescent period/adulthood, or it can include both present and remote consequences [1]. This study will provide new insight into oral health-related behaviors: smoking should be viewed as one of them, for it has deeper connections with other behaviors and might have a direct infects on OHRQoL.

Our study verified the Problem Behavior Theory. Firstly, the theory suggested that the problem behaviors were positively associated in both samples. People who smoke during adolescence are more likely to consume SSB sand have poor oral hygiene habits. Some evidence also reveals that smoking consumption in adolescents can be associated with the intake of SSBs [55]. Even in adults, a study about maternal smoking showed that mothers who reported consuming >1 cup of soft drink per day were more likely to smoke than those consuming fewer soft drinks [56]. Secondly, in the social-psychological framework of the theory, various problem behaviors correlated similarly with a number of personalities and social environment variables [1]. In this study, respondents’ gender and their parents’ level of education were variables affecting all three behaviors. In our study, boys and adolescents with less-educated fathers had a higher risk of smoking, SSBs, and poor oral hygiene habits. A study in Japan also found that parental education was related to child health-related behaviors [57]. Meanwhile, the social-economic status of parents [58], peer influence [59], home/school environment, and other surrounding environments [60] might also be co-influencing factors. According to the Problem Behavior Theory, adolescents’ problems are socially constructed and result from interactions between individual characteristics and social environments. Such problems are determined by a balance of instigations and controls across three systems, including the perceived environmental system (e.g., social controls and supports), personality system (e.g., values, expectations, and beliefs), and behavior system [61]. Studies are needed to explore these systems and the interactions of brushing teeth, SSB consumption, and smoking. Furthermore, studies regarding oral health disparities among adolescents should carefully choose an SES indicator and other factors, considering multiple pathways between these indicators and health/health behaviors.

The results of this study suggest that intervention and change efforts should not focus only on specific behaviors, but combine all related behaviors, and figure out connections and internal mechanisms among them. Only in this way could we provide substantive programs for oral health prevention. Normally, oral public health professionals focus their efforts on tooth-brushing education and public clinical intervention promotion. More studies could be designed to find out the problems these behaviors can bring and the meanings they represent, and implement interventions on the lifestyles of adolescents. We conclude that the clustering of health behaviors among adolescents suggests that a more global/national approach to behavior change may be necessary. Considering cultural differences, the findings in the study warrant further study in other counties.

## 5. Limitations

There are several limitations to this study. First, the cross-sectional design prohibits causal inferences; the study was conducted in one province, and the smoking sample was only 119, which does not represent the whole population. Because OHRQoL was valued by a simplified Chinese version of COHIP, the information may not be complete. Selection bias might also exist for smokers, including those who only tried smoking once. Furthermore, SSB Consumption was valued by fluency, the quantity and components should be included.

## 6. Conclusions

Three behaviors should be seen as oral health-related behaviors, adolescents’ health risk behaviors, or problem behavior syndrome. The co-variations among the three behaviors were gender and parents’ education level. After adjusting for demographic factors, smoking adolescents would have a higher level of soft drink consumption, worse oral hygiene habits, and poorer OHRQoL. The results suggest that more studies should be designed to find out the problems these behaviors have brought and the meanings they represent, and to figure out the connections and internal mechanisms among the behaviors to provide substantive programs for oral health prevention. Oral public health personnel should redouble their efforts on behavior intervention and related implements on adolescents’ lifestyles.

## Figures and Tables

**Table 1:** The questionnaire used in the study.

Social Demographic Characteristics	
Gender	(1) Male (2) Female
Birthday	Year___Month___Day____
Ethnicity	(1) Han nationality (2) Minor nationality
Are you the only child in the family?	(1) Yes (2) No
The highest education level of your father is?	(1) Never go to school (2) Primary school Middle school (3) High school Technical school (4) Technical secondary school (5) Junior college Graduate ofthe university and above (6) Don’t have father or don’t know
The highest education level of your mother is?	(1) Never go to school (2) Primary school Middle school (3) High school Technical school (4) Technical secondary school (5) Junior college Graduate ofthe university and above (6) Don’t have mother or don’t know
Do you brush your teeth?	(1) Yes, often (2) Occationally or never
How often do you brush your teeth?	(1) Twice or more per day (2) Once per day (3) Do not brush teeth every day
Do you smoke?	(1) Yes, every day (2) Yes, every week (3) Yes, but seldom or smoked before (4) No, never
How often do you drink soft drinks (including soda drinks like cola, fruit juice like orange juice)?	(1) Twice or more per day (2) Once per day (3) 2–6 times per week (4) Once per week (5) 1–3 times per month (6) Seldom or never
How often do you drink sugary milk, yogurt, milk powder, tea, soybean, and coffee?	(1) Twice or more per day (2) Once per day (3) 2–6 times per week (4) Once per week (5) 1–3 times per month (6) Seldom or never
How greatly has it affected you during the past six months because of your oral problems	
Eating	
Pronouncing words	(1) Sever (2) Median (3) Minor (4) None
Brushing teeth	(1) Sever (2) Median (3) Minor (4) None
Doing housework	(1) Sever (2) Median (3) Minor (4) None
Going to school	(1) Sever (2) Median (3) Minor (4) None
Sleeping	(1) Sever (2) Median (3) Minor (4) None
Smiling	(1) Sever (2) Median (3) Minor (4) None
Easily being worried	(1) Sever (2) Median (3) Minor (4) None
Social communication	(1) Sever (2) Median (3) Minor (4) None

**Table 2:** Smoking prevalence by demographics characteristics, tooth brushing.

Variables	*n*	% of Sample	Smoking Prevalence (%)	
			tt/χ^2^	*p* Value
**Age**			6.21	0.102
12	1349	21.4	1.3	
13	1675	26.5	1.9	
14	1615	25.6	1.7	
15	1672	26.5	2.5	
**Gender**			18.71	<0.001 **
Male	3141	49.8	2.6	
Female	3170	50.2	1.1	
**Ethnicity**			0.93	0.340
Han	6135	97.2	1.8	
Minority	176	2.8	2.8	
**Place of residence**			4.19	0.041 *
Urban	3155	50.0	2.2	
Rural	3156	50.0	1.5	
**Only Child**			1.67	0.196
Yes	2830	44.8	1.7	
No	3481	55.2	2.1	
**Highest education level of father**			11.63	0.009 **
Elementary school or less	1000	15.8	2.9	
Junior high school	2839	45.0	2.1	
High school	1123	17.8	1.0	
Junior college, college or higher	396	6.3	1.3	
**Highest education level of mother**			9.96	0.019 *
Elementary school or less	1417	22.5	2.8	
Junior high school	2687	42.6	1.5	
High school	929	14.7	1.3	
Junior college, college or higher	335	5.3	2.4	
**Frequency of tooth brushing**			13.04	0.001 **
Less than once per day	1283	20.3	3.0	
Once per day	2428	38.5	1.8	
Twice or more per day	2600	41.2	1.4	
**Frequency of soft drink consumption**			29.38	<0.001 **
Once or more per day	1235	19.6	3.7	
Twice to 6 times per week	1592	25.2	1.6	
Once or less per week	3484	55.2	1.3	
**Frequency of drinking milk, tea, coffee with sugar**			7.29	0.026 *
Once or more per day	2201	34.9	2.5	
Twice to 6 times per week	1679	26.6	1.5	
Once or less per week	2431	38.5	1.5	
**Oral Health quality of life**	11	9.15	−2.68	0.007 **

* *p* < 0.05 ** *p* < 0.001.

**Table 3:** Demographics characteristics of participants by tooth brushing and SSB consumption frequency.

Variables	Tooth Brushing (%)			Soft Drink Consumption (%)			Drinking Milk, Tea, Coffee with Sugar (%)		
	<1/d	=1/d	≥2/d	≤1/W	2–6/W	≥1/d	≤1/W	2–6/W	≥1/d
**Age**	χ^2^ = 68.02 *p* < 0.001 **			χ^2^ = 13.86 *p* = 0.031 *			χ^2^ = 3.79 *p* = 0.705		
12	18.9	40.0	41.1	58.9	22.8	18.3	39.3	26.8	33.9
13	17.0	39.3	43.7	55.3	26.1	18.6	38.4	25.5	36.1
14	18.8	42.4	38.8	53.5	25.1	21.4	38.3	27.9	33.9
15	26.3	32.7	41.1	53.8	26.4	19.9	38.2	26.3	35.5
**Gender**	χ^2^ = 190.22 *p* < 0.001 **			χ^2^ = 192.77 *p* < 0.001 **			χ^2^ = 1.69 *p* = 0.429		
Male	23.7	43.6	32.6	47.0	28.0	25.1	37.9	26.5	35.6
Female	17.0	33.3	49.7	63.4	22.5	14.1	39.1	26.7	34.1
**Ethnicity**	χ^2^ = 29.00 *p* < 0.001 **			χ^2^ = 6.89 *p* = 0.032 *			χ^2^ = 0.44 *p* = 0.803		
Han	19.9	38.5	41.6	55.4	25.2	19.4	38.6	26.6	34.8
Minority	35.2	38.1	26.7	47.2	26.1	26.7	36.4	26.7	36.9
**Place of residence**	χ^2^ = 150.80 *p* < 0.001 **			χ^2^ = 2.61 *p* = 0.271			χ^2^ = 0.58 *p* = 0.749		
Urban	25.3	31.9	42.8	56.2	24.5	19.3	39.0	26.4	34.6
Rural	15.4	45.0	39.6	54.2	25.9	19.9	38.1	26.8	35.1
**Only Child**	χ^2^ = 15.31 *p* < 0.001**			χ^2^ = 10.50 *p* = 0.005 **			χ^2^ = 0.89 *p* = 0.641		
Yes	18.2	39.9	41.9	53.0	26.3	20.7	38.4	26.1	35.4
No	22.1	37.3	40.6	57.0	24.3	18.6	38.6	27.0	34.4
**Highest education level of father**	χ^2^ = 69.50 *p* < 0.001 **			χ^2^ = 14.5 *p* = 0.024 *			χ^2^ = 20.74 *p* = 0.002 **		
Elementary school or less	18.6	45.5	35.9	54.2	26.0	19.8	41.9	27.9	30.2
Junior high school	18.5	40.2	41.3	53.5	26.0	20.5	38.0	26.7	35.3
High school	20.1	34.6	45.3	57.3	24.5	18.2	37.2	26.4	36.4
Junior college, college or higher	24.2	23.7	52.0	62.4	22.0	15.7	34.8	23.0	42.2
**Highest education level of mother**	χ^2^ = 55.76 *p* < 0.001 **			χ^2^ = 10.02 *p* = 0.124			χ^2^ = 15.85 *p* = 0.015 *		
Elementary school or less	19.8	43.3	36.8	53.9	25.6	20.5	39.4	29.1	31.4
Junior high school	18.2	39.3	42.5	56.4	24.8	18.8	38.1	26.1	35.7
High school	20.8	31.9	47.4	54.7	26.3	19.1	39.7	25.0	35.3
Junior college, college or higher	21.8	27.5	50.7	62.7	21.2	16.1	33.1	26.9	40.0

* *p* < 0.05. ** *p* < 0.001.

**Table 4:** Multiple logistic regression analysis by adjusting demographics factors.

Variables	OR (95% CI)			
	Model 1	Model 2	Model 3	Full Model
**Frequency of tooth brushing**				
Less than once per day	1.89 (1.13–3.17) *			1.74 (1.03–2.93) *
Once per day	1.11 (0.68–1.83)			1.07 (0.65–1.76)
Twice or more per day (ref)	1			1
**Frequency of sweet soft drink consumption**				
Once or less per week		2.62 (1.63–4.20) **		2.18 (1.32–3.60) **
Twice to 6 times per week		1.32 (0.78–2.23)		1.26 (0.74–2.16)
Once or more per day (ref)		1		1
**Frequency of sugary beverage consumption**				
Once or less per week			1.96 (1.22–3.15) **	1.52 (0.92–2.50)
Twice to 6 times per week			1.12 (0.64–1.97)	1.04 (0.59–1.85)
Once or more per day (ref)			1	1

OR: odd ratio; CI: confidence interval; * p < 0.05. ** *p* < 0.001.

**Table 5:** Frequency distribution of responses for the items of COHIP.

Items of COHIP	Non-Smoker (%)				Smoker (%)				χ^2^	*p* Value
	None	Minor	Median	Sever	None	Minor	Median	Sever		
Eating	39.3	31.1	19.3	10.3	33.3	35.0	18.8	12.8	2.3	0.508
Pronouncing words	78.4	13.9	6.2	1.6	66.1	17.9	11.6	4.5	14.4	0.002 **
Brushing teeth	59.0	21.3	12.5	7.2	58.2	15.5	16.4	10.0	4.1	0.248
Easily being worried	67.7	18.2	9.0	5.1	57.8	24.8	5.5	11.9	15.2	0.002 **
Sleeping	73.6	15.3	6.9	4.3	62.0	21.3	7.4	9.3	10.5	0.015 *
Smiling	65.5	18.9	9.0	6.6	56.0	19.3	10.1	14.7	12.2	0.007 **
Social communication	77.5	12.8	6.0	3.6	61.3	17.0	11.3	10.4	22.5	<0.001 **
Go to school	80.3	12.0	5.2	2.5	70.3	17.1	5.4	7.2	13.2	0.004 **
Doing housework	93.5	4.7	1.3	0.5	89.7	7.5	2.8	0.0	4.1	0.203

* *p* < 0.05. ** *p* < 0.001.

## Data Availability

The data presented in this study are available on reasonable request from the corresponding author. The data are not publicly available due to the participants’ privicy.
